# Egg Overactivation—An Overlooked Phenomenon of Gamete Physiology

**DOI:** 10.3390/ijms26094163

**Published:** 2025-04-27

**Authors:** Alexander A. Tokmakov, Ken-Ichi Sato

**Affiliations:** 1 Faculty of Biology-Oriented Science and Technology, KinDai University, 930 Nishimitani, Kinokawa City 649-6493, Japan; 2 Faculty of Life Sciences, Kyoto Sangyo University, Kamigamo-Motoyama, Kita-ku, Kyoto 603-8555, Japan; kksato@cc.kyoto-su.ac.jp

**Keywords:** *Xenopus laevis*, eggs, activation, overactivation, apoptosis, necrosis

## Abstract

In many vertebrates, mature ovulated eggs are arrested at metaphase II prior to fertilization. The eggs exit meiotic arrest after fertilization-induced or parthenogenetic activation, followed by embryo development or egg degradation, respectively. Calcium-dependent activation of meiotically-arrested eggs has been thoroughly investigated in various species. In addition, several recent studies have detailed the excessive activation of ovulated frog eggs, so-called overactivation. This overview highlights the major events of overactivation observed in mature ovulated eggs of the African clawed frog *Xenopus laevis* with a focus on similarities and differences between spontaneous, oxidative stress-induced, and mechanical stress-induced overactivation. The dramatically different cell death scenarios that unfold in activated and overactivated eggs are also exposed in the article.

## 1. Introduction

Ovulated fertilization-competent eggs from many vertebrate species are arrested at the metaphase of the second meiotic division prior to fertilization. The meiotic metaphase arrest prevents cell cycle progression and parthenogenesis. Meiotically arrested eggs that remain unfertilized for an extended period grow older and sustain various injuries, resulting in the loss of their quality. Infertility in various animals, including mammals, can be attributed to poor-quality oocytes and eggs [1,2]. Reduced rates of fertilization, polyspermy, parthenogenesis, and aberrant embryonic development are caused by age- and stress-induced damage. As a result of this damage, unfertilized mature eggs gradually deteriorate over time and eventually die. Notably, in many cases, eggs from different species have been found to die by apoptosis in the absence of fertilization [3]. Fragmentation of ovulated murine oocytes [4], calcium-triggered degradation of rat eggs [5], aging of unfertilized sea urchin eggs [6,7], and spontaneous deterioration of mature unfertilized frog eggs [8,9,10] are the well-described examples of caspase-mediated apoptotic cell death. Although apoptosis may represent a major and by far predominant mechanism of cell death in mature unfertilized eggs, it seems that non-apoptotic scenario(s) can also be executed in these cells. It was recently demonstrated that the excessive activation of ovulated frog eggs, so-called overactivation, triggers necrotic cell death [11]. Notably, although overactivation followed by necrotic cell death can be artificially induced by a strong activation stimulus, such as oxidative or mechanical stress, it also occurs spontaneously, albeit with a low frequency, usually not exceeding 2%, in natural populations of frog eggs [11]. The intracellular events of egg overactivation and following cell death unfold progressively in a sequential manner, suggesting an ordered physiological process. In this review, we describe the major events of spontaneous and stress-induced overactivation and following necrotic cell death observed in mature unfertilized eggs of the African clawed frog *Xenopus laevis.* The two notably different cell death scenarios that unfold in activated and overactivated eggs are also highlighted in the paper.

## 2. Egg Maturation and Metaphase II Arrest

**Figure 1:**
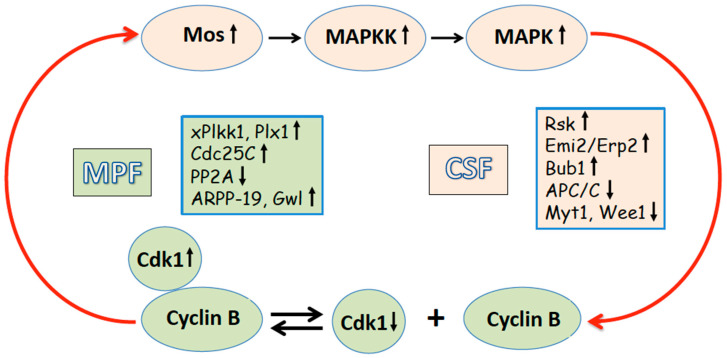
Meiotic metaphase arrest in *Xenopus* eggs is maintained by active MPF and CSF. The molecular components of MPF and CSF are colored green and pink, respectively. MPF and CSF are embedded in a loop of positive feedback (red arrows). Black upward and downward arrows next to the components of CSF and MPF indicate their activated and inhibited states, respectively.

In all sexually reproducing animals, including the established developmental model, African clawed frog, *Xenopus laevis*, oocytes undergo meiotic reduction divisions in order to produce eggs with a haploid chromosomal content. Immature, fully-grown, and fertilization-incompetent *Xenopus* oocytes reside in the frog ovaries while arrested at the diplotene stage of the first meiotic prophase. The immature oocytes exhibit the minimal activity of the key meiotic regulators, the maturation promoting factor (MPF, a complex of cyclin B and Cdk1 kinase) and cytostatic factor (CSF, a multi-component signaling system involving the meiotic protein kinase Mos and the MAPK pathway) (Figure 1). MPF was originally defined as the cytoplasmic activity from eggs that causes complete maturation upon injection into immature oocytes, and CSF is a factor that causes metaphase arrest in frog eggs [12,13]. Of note, the essential component of CSF, the Mos protein, is only present during meiosis and vanishes following fertilization [14]. A portion of Cdk1 is stored in an inactive complex known as pre-MPF, while the majority of Cdk1 in immature oocytes is present in a free, inactive monomeric state. The catalytic activity of Cdk1 in the oocytes is inhibited by phosphorylation on Thr 14 and Tyr 15 by the inhibitory kinase Myt1 [15]. Wee1, another identified Cdk1-inhibitory kinase, begins to accumulate near the meiotic I exit and is not expressed in immature *Xenopus* oocytes [16,17]. In addition, direct phosphorylation on Ser 287 inhibits the MPF-activating phosphatase Cdc25C [18].

In frogs, the steroid hormone progesterone, which is produced by the follicle cells surrounding oocytes, triggers the meiotic maturation of oocytes while they are still in the ovaries. During maturation and meiotic resumption, oocytes go through the meiotic cell cycle and pause again after ovulation. In many species, including mammals, frogs, and fishes, mature eggs pause at the metaphase of the second meiotic division prior to fertilization. In frogs, the term “egg” refers to the ovulated mature oocytes arrested in metaphase II. The eggs are halted in metaphase II because of the high activity of MPF and CSF. Cyclin B, accumulated during maturation, directly binds and activates Cdk1 kinase. Meanwhile, the high activity of the MAPK cascade is supported by the newly synthesized Mos protein. Meiotic arrest prevents the progression of parthenogenetic mitotic cell cycles after meiosis, allowing eggs to wait for fertilization. Importantly, CSF and MPF are crucially involved in a positive feedback loop (Figure 1). Active Cdk1/cyclin B enhances the stability of the Mos protein by its direct phosphorylation on Ser 3 [19]. Furthermore, de novo Mos synthesis is boosted by cytoplasmic polyadenylation of maternal mos mRNA mediated by the MAPK signaling cascade [20]. In turn, Mos activates MAPK, which phosphorylates and activates the downstream target protein kinase Rsk. Active Rsk then phosphorylates and downregulates Myt1, the Cdk1-inhibitory kinase [15,21]. Wee1, another Cdk1-inactivating kinase, is likewise suppressed in metaphase-arrested eggs through a phosphorylation-dependent mechanism [22]. Moreover, active Rsk phosphorylates and activates the inhibitors of the APC/C ubiquitin ligase, Emi2/Erp1 and Bub1 proteins, thereby suppressing cyclin B degradation [23,24,25]. Emi2/Erp1 protein is not expressed in prophase oocytes, but it accumulates in mature metaphase-arrested eggs due to cytoplasmic polyadenylation and translational unmasking of its mRNA [26]. This protein was recognized as a pivotal component of CSF required to maintain meiotic metaphase arrest [27,28]. The phosphorylated inhibitor proteins sequester the ligase’s activator component Cdc20, hence downregulating APC/C [29]. In addition, Cdc20 is sequestered by the spindle assembly checkpoint (SAC) proteins, such as MAD2, BUB3, and BUBR1. In metaphase I, SAC controls kinetochore–microtubule occupancy and is activated through a response to unoccupied kinetochores, preventing APC/C activation and anaphase onset [30]. Aurora kinase B was found to activate SAC by triggering depolymerization of unbound microtubules [31,32]. Furthermore, *Xenopus* polo-like kinase kinase xPlkk1 and polo-like kinase Plx1 are two components of the polo-like protein kinase pathway that are activated by both Cdk1 and MAPK [33,34]. The upregulation of the MPF-activating phosphatase Cdc25C is associated with this pathway [35,36]. High MPF activity in eukaryotic meiotic eggs and mitotic somatic cells was found to be associated with the inhibition of the prime anti-Cdk1 phosphatase PP2A-B55, which is responsible for dephosphorylating Cdk1-phosphorylated substrates [37].

The Greatwall kinase (Gwl), which is activated downstream of Cdk1/cyclin B, mediates this event [38,39,40]. Gwl phosphorylates two small proteins, ARPP-19 and/or a-endosulfine, which are around 20 kDa in size. These proteins then bind with and suppress PP2A-B55 phosphatase [41,42]. The Gwl/ARPP19/PP2A-B55 module was shown to play a significant role in the MPF auto-amplification loop, contributing to the activation of MPF during the maturation of *Xenopus* oocytes [43,44]. High MPF activity is also maintained by other mechanisms, such as the Mos pathway-stimulated polyadenylation of cyclin mRNA [45].

## 3. Egg Activation and Exit from the Meiotic Metaphase Arrest

Therefore, several interlocking feedback loops contribute to the stability of metaphase II arrest in mature eggs before fertilization, and the active MPF and CSF are responsible for maintaining the arrest (Figure 1). Fertilization causes the disruption of the positive feedback between MPF and CSF and their inactivation via calcium-dependent mechanisms. Markedly, the sperm-triggered calcium transient universally activates eggs and releases them from the meiotic arrest, even though eggs from different species may be arrested at different stages of the meiotic cell cycle prior to fertilization, as reviewed in [46,47]. In every sexually reproducing animal that has been investigated, calcium acts as a universal messenger to mediate egg activation at fertilization. However, there are significant species-to-species differences in the mechanisms of calcium-induced exit from meiotic arrest and the pathways that generate the intracellular calcium signal. An increase in intracellular calcium is necessary and sufficient for egg activation; calcium ionophores can induce egg activation in the absence of fertilization. Markedly, extracellular calcium was found to be involved in the activation of eggs from several marine species, including echinoderms, marine worms, etc., as reviewed in [48,49]. In addition, calcium influx-mediated signaling was shown to be required for the complete activation of mouse eggs [50]. Store-operated calcium entry and transient receptor potential channels have been pinpointed as the driving forces of calcium influx during activation of mammalian eggs, as reviewed in [51]. However, in frogs, the release of calcium from intracellular calcium stores, such as the endoplasmic reticulum, is primarily responsible for the initial elevation of cytosolic calcium in activated eggs, and the following self-propagation of the calcium signal involves intracellular calcium-induced calcium release [52,53,54]. Various parthenogenetic factors, such as mechanical stress, oxidative stress, electric shock, etc., can activate eggs through the elevation of intracellular calcium. Also, spontaneous egg activation, which has been implicated as a major factor of fertilization incapacity, was found to involve calcium-dependent mechanisms in mammalian eggs [55,56]. Notably, calcium-independent mechanisms can also be involved in the spontaneous activation of aging eggs. The gradual inactivation of the MAPK pathway and/or MPF has been suggested to trigger spontaneous activation of aging unfertilized sea urchin eggs [57,58,59].

**Figure 2:**
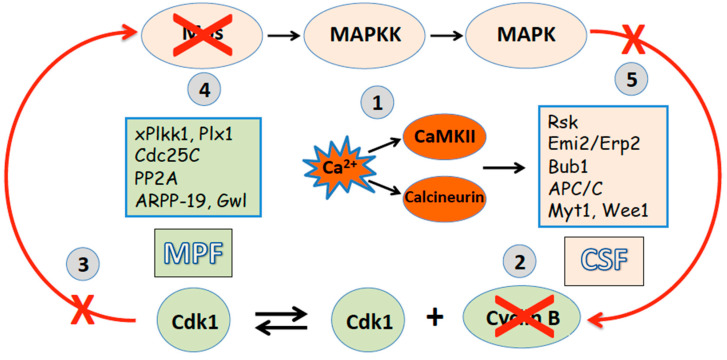
Egg activation downregulates MPF and CSF, resulting in meiotic exit. A calcium-mediated activation signal (1) stimulates APC/C, leading to the degradation of cyclin B (2), inactivation of MPF (3), destabilization of Mos protein (4), loss of CSF activity (5), and exit from meiotic metaphase II arrest. Red arrows indicate a loop of positive feedback between MPF and CSF.

Thus, generally, the intracellular calcium signal represents a key early event of both fertilization-induced and parthenogenetic egg activation, which triggers a cascade of calcium-dependent processes resulting in meiotic exit (Figure 2). In frog eggs, the calcium signal independently activates the calcium/calmodulin-dependent protein kinase, CaMKII, and the calcium/calmodulin-dependent serine/threonine protein phosphatase calcineurin, PP2B. CaMKII directly phosphorylates the inhibitor of the APC/C ubiquitin ligase, Emi2/XErp1, fostering the formation of a phosphorylation-dependent degradation signal. This signal is then recognized by the SCF1 (SKP2-cullin1-F-box protein)-E3 ubiquitin ligase complex, which targets Emi2 for 26S proteasome-mediated destruction [60,61]. The destruction of Emi2/XErp1 leads to the activation of the APC/C ubiquitin ligase, resulting in the ubiquitination of cyclin B and its subsequent degradation by the 26S proteasome, as reviewed in [62,63]. In parallel, calcineurin dephosphorylates Apc3, the core APC/C component, and the APC/C activator Cdc20, supporting APC/C activation [64]. As a result of the proteasome-dependent degradation of cyclin B by the two independent mechanisms, Cdk1 activity is inhibited, and MPF becomes inactive in fertilized frog eggs. Consequently, Cdk1 inactivation disrupts the positive feedback loop between MPF and CSF [65] (Figure 2). The Mos protein becomes dephosphorylated at the Serine 3 site of direct phosphorylation by Cdk1 and then degraded by the N-terminal proline-dependent ubiquitin pathway [66]. The breakdown of Mos results in the shutdown of the MAPK cascade, CSF inactivation, and exit from meiosis. The response of additional factors that regulate meiotic metaphase II arrest, such as Myt1 and Wee1 kinases, Cdc25C phosphatase, polo-like protein kinases, Greatwall kinase, etc., has been discussed more extensively elsewhere [67]. Markedly, the participation of CaMKII and calcineurin in egg activation is not conserved throughout evolution. The eggs of many invertebrate species employ the “phosphatase-only” mechanism of meiotic exit because their genomes do not encode the CaMKII target protein Erp1 [68]. On the contrary, the exit from meiotic metaphase arrest and cell cycle resumption in mammalian eggs were found to depend entirely on CaMKII activity [69,70].

## 4. Egg Overactivation, Its Inducers and Hallmarks

The processes of egg activation and meiotic exit described above have been extensively investigated in various species. In addition, several recent studies have provided detailed information about the excessive activation of mature ovulated frog eggs, which was referred to as overactivation. Then, what are the major features of overactivation that distinguish it from normal activation? Of note, egg overactivation can occur spontaneously, or it can be triggered by strong extracellular stimuli, such as oxidative or mechanical stress [Table 1]. The main events of spontaneous and stress-initiated overactivation are essentially the same; however, minor differences exist between these processes, as discussed further.

One of the most prominent visually observed events of overactivation is the irreversible contraction of the egg cortical layer that gives rise to a very distinctive and easily recognizable egg phenotype. In contrast to the reversible calcium/protein kinase C-mediated and actin/myosin-based cortical contraction observed in fertilized or parthenogenetically activated *Xenopus* frog eggs, the cortical contraction in overactivated eggs is irreversible, resulting in progressive egg whitening. The cortical contraction proceeds very quickly in the overactivated eggs, and these cells nearly turn white within an hour [11,71,72]. The phenomenon of irreversible cortical contraction can be explained by the uncompensated increase in intracellular calcium that takes place in overactivated eggs. It was found that intracellular calcium levels remain consistently high during oxidative stress-induced *Xenopus* egg overactivation, unlike in fertilization-induced egg activation, where the calcium transient only lasts for a few minutes [73]. In this context, the treatment of frog eggs with the calcium ionophore A23187 was shown to trigger egg activation without fertilization [74]. However, it was noted in various species that the calcium transient generated in eggs by calcium ionophores is different from the physiological signal; the ionophores can induce only a single broad signal, but not the species-specific patterns observed during fertilization. For instance, in mammals, calcium ionophores produce a single peak followed by a slow decline over several minutes [49]. This pattern does not mimic fertilization-triggered calcium oscillations, and it results in abnormal egg activation. It is interesting to investigate if the excessive ionophore treatment, inducing the protracted elevation of intracellular calcium levels, would lead to frog egg overactivation, similar to prolonged oxidative stress.

The degradation of meiotic cyclin B is another universal feature observed in overactivated frog eggs. Overactivation was found to promote robust degradation of M phase-specific cyclin B, which maintains high MPF activity in the metaphase of the second meiotic division [11,72]. Cyclin B is rapidly degraded both in the eggs overactivated by mechanical stress and in spontaneously overactivated eggs (Table 1). As in fertilized or parthenogenetically activated eggs, cyclin B degradation can be detected in overactivated eggs as soon as 30 min after triggering overactivation by oxidative stress [72]. As discussed above, cyclin degradation in activated frog eggs is caused by the elevation of intracellular calcium and is mediated by CaMKII and calcineurin.

The rapid depletion of intracellular ATP is a prominent characteristic of overactivation. It has been observed in eggs overactivated either spontaneously or by mechanical or oxidative stress [Table 1]. Even though parthenogenetic egg activation also causes a notable and significantly delayed decrease in intracellular ATP [8], the change observed in the overactivated eggs is extremely rapid and drastic. The ATP decline becomes evident 30 min after inducing egg overactivation, and almost complete depletion of intracellular ATP occurs in the eggs within 1 h [11,71]. The dynamics of intracellular ATP reflect very different cell death scenarios unfolding in activated and overactivated eggs (see next section for details). Large quantities of leaked ATP have been detected outside overactivated eggs, suggesting that plasma membrane permeability is substantially elevated in overactivated eggs, permitting the release of intracellular ATP [11]. Given the remarkably robust ATP discharge, it is reasonable to suggest that plasma membrane integrity is likely compromised in these cells. Moreover, the ADP/ATP ratio is significantly elevated in overactivated *Xenopus* eggs [Table 1] and in their extracellular compartment [11]. These findings indicate that overactivated eggs lose their ATP not only through leakage but also through the intracellular conversion of ATP to ADP, which is also discharged from the eggs. A decline in MMP was witnessed in the eggs overactivated by oxidative stress [72], suggesting that ATP synthesis might be inhibited in these cells. It should be noted, however, that overactivation was induced by unphysiologically high concentrations of hydrogen peroxide in these studies. The rapid depletion of intracellular ATP can explain the termination of protein synthesis and the lack of caspase activation in overactivated eggs (Table 1) [11,72].

Indeed, protein synthesis is one of the most energy-consuming intracellular processes that requires both ATP and GTP for its execution. Inhibition of protein synthesis leads to a gradual decrease in soluble cytoplasmic protein content and progressive disruption of protein homeostasis in the eggs overactivated by oxidative stress (Table 1) [71]. As with protein synthesis, caspase-dependent apoptosis requires high levels of ATP for its execution [75,76]. Specifically, apoptosome assembly, which represents the major step in caspase activation, depends on the presence of cytochrome C and ATP/dATP binding. A reduction in intracellular ATP occurs quite late in apoptosis because high ATP levels are required to sustain this process. 

The other universal and visually recognizable event of overactivation is egg swelling, which can be detected within 20 min of triggering overactivation [11,72]. The rapid and significant increase in the cell size suggests that membrane permeability is greatly increased and that osmotic homeostasis is critically damaged in overactivated eggs. The leakage of large amounts of ATP and ADP from the overactivated eggs, as described above, indicates that plasma membrane integrity is compromised in these cells. Apparently, the uptake of extracellular liquid caused by the plasma membrane damage can explain the swelling of overactivated eggs. Still, the physical damage of the plasma membrane does not lead to the immediate massive release of intracellular content; overactivated eggs retain their shape and size for some time. Notably, plasma membrane rupture conventionally identifies necrotic cell death, as defined by the Nomenclature Committee on Cell Death [77]. The following section describes the cell death scenarios unfolding in overactivated and activated eggs.

It appears that there are still distinctions between stress-induced and spontaneous overactivation. For example, the amount of leaked ATP is greater, and the ADP/ATP ratio in both extracellular and intracellular components is lower in eggs overactivated by mechanical stress, as compared to spontaneously overactivated eggs [11]. The greater damage to the plasma membrane from mechanical stress can account for these observations, causing ATP to leak more quickly before it can be converted to ADP by intracellular enzymes. Moreover, while cyclin B is degraded both in eggs overactivated by mechanical stress and spontaneously overactivated eggs, MAPK is exclusively dephosphorylated only in the latter scenario (Table 1) [11]. This difference can also be explained by the variation in the levels of plasma membrane damage. A positive feedback loop operates between MPF and CSF in mature eggs to maintain meiotic arrest before fertilization (Figure 1) [78]. Furthermore, cyclin degradation was found to occur prior to the downregulation of the MAPK pathway in activated *Xenopus* eggs [67,79]. It can be suggested that the mild breach of the plasma membrane in spontaneously overactivated eggs maintains the positive feedback loop between MPF and CSF for a long enough time to initiate CSF inactivation and MAPK dephosphorylation. However, the membrane undergoes more severe damage in stress-overactivated eggs, leading to quick disruption of this feedback and thereby preventing MAPK dephosphorylation. Thus, the observed minor differences between spontaneous and stress-induced overactivation can be attributed to varying degrees of damage to the plasma membrane. Besides, some of the overactivation features can only be observed in the eggs overactivated by a specific stressor. For instance, lipofuscin accumulation and intracellular acidification take place in the eggs overactivated by hydrogen peroxide (Table 1). However, these phenomena can hardly be expected to occur in the eggs activated spontaneously or by mechanical stress. As hydrogen peroxide is known to induce oxidation of proteins and lipids, it is conceivable that the content of lipofuscin, a nondegradable aggregate of oxidized lipids, proteins, and metals, increases in peroxide-treated eggs [71]. In addition, the weak acidity of hydrogen peroxide and its high permeability may be related to intracellular acidification of the eggs overactivated by this drug (Table 1).

## 5. Different Fates of Activated and Overactivated Eggs

**Figure 3:**
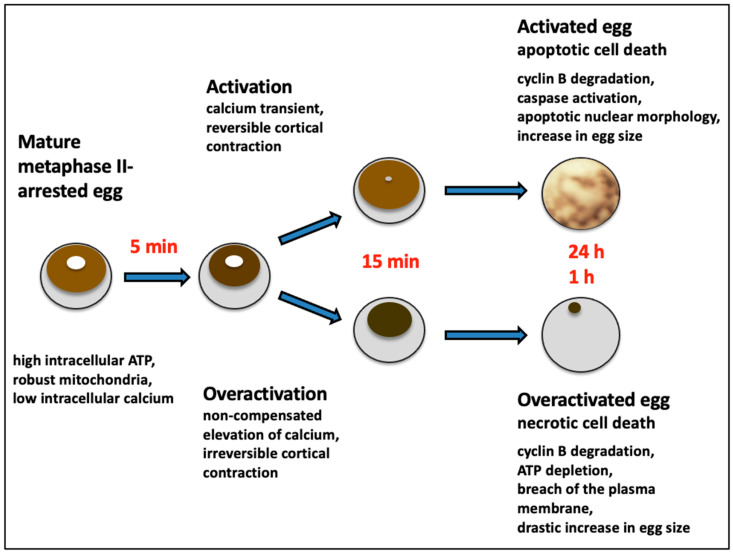
Different cell death scenarios in activated and overactivated eggs.

Although many features of egg overactivation and activation, such as the elevation of intracellular calcium, cortical contraction, the degradation of M phase-specific cyclin B, etc., are similar, the cell death scenarios that unfold in these cells are dramatically different. A detailed comparison of the intracellular molecular events of egg activation and overactivation is beyond the scope of this paper; this will be discussed elsewhere. Here, we would like to make a clear distinction between the two different modes of cell death following egg overactivation and activation and highlight the features that establish egg overactivation as a unique and largely overlooked phenomenon of gamete physiology (Figure 3).

The process that develops in the aging *Xenopus* eggs after their parthenogenetic activation has been identified as apoptosis by several studies. This assignment is based on the appearance of the hallmark features of the classical apoptotic process, such as the involvement of proapoptotic Bcl-2 family proteins, the release of cytochrome C from mitochondria, activation of caspases, apoptotic nuclear morphology, decline in intracellular ATP, etc. [8,9,10]. These features are accompanied by clearly visible changes in egg morphology, such as gradual decoloring and swelling of the eggs. Apoptosis in activated frog eggs is quite slow; it unfolds gradually over 48 h following activation. A great majority of unfertilized eggs are degraded by this process; thus, apoptosis should be considered as a default cell death scenario for unfertilized frog eggs.

On the other hand, overactivation and following cell death affect only a small minority of unfertilized eggs, normally not exceeding 1–2%. Overactivation cannot be observed at all in some populations of highly stable eggs laid by young, healthy animals. The process that unfolds in *Xenopus* eggs after spontaneous or stress-induced overactivation is extremely robust; its main intracellular events develop within an hour (Figure 3). Also, the morphology of overactivated eggs changes very rapidly, and within just one hour, these cells turn nearly completely white (see previous section for details). As detailed in the previous section, the irreversible cortical contraction is caused by an uncompensated rise in intracellular calcium levels in overactivated eggs. The cell death scenario in overactivated eggs has recently been categorized as necrosis [11,72]. This type of cell death is characterized by the virtually instantaneous physical disassembly of the plasma membrane. The finding that ATP and ADP are released extensively from overactivated frog eggs [11] indicates that the plasma membrane is damaged in these cells. The phenomenon of ATP depletion was consistently observed in the eggs overactivated by oxidative or mechanical stresses [71] or in those overactivated spontaneously (Table 1) [11,72]. After triggering overactivation, it takes about one hour to deplete intracellular ATP to a trace level. Furthermore, the rapid termination of protein synthesis in the overactivated eggs is caused by the depletion of intracellular ATP, as detailed in the previous section.

The rapid loss of intracellular ATP is a hallmark of classical necrosis that distinguishes it from other types of cell death [75], such as apoptosis and autophagy, because these processes require ATP for their execution. It was proposed that intracellular levels of ATP dictate a particular mode of cell death through necrosis or apoptosis [80], and it was subsequently shown that ATP depletion can alter the mode of cell death [81,82]. Notably, overactivated frog eggs experience ATP loss, not only from leakage but also from intracellular conversion of ATP to ADP [11]. In the past, the rapid metabolic depletion of ATP has been seen in different types of somatic cells undergoing necrotic cell death [83]. It was reported that necrosis causes mitochondrial dysfunction due to inner membrane depolarization, leading to a decrease in ATP production [84]. In our studies, a decrease in MMP was observed in the eggs overactivated by oxidative stress (Table 1) [72], suggesting that ATP synthesis is inhibited in these cells.

A significant increase in cell size was evident in frog eggs within just 20 min of triggering overactivation [11,72]. Apparently, it is the loss of plasma membrane integrity that accounts for this phenomenon. The increase in membrane permeability and disruption of osmotic homeostasis in overactivated eggs should be the outcome of membrane damage. Of note, an increase in cell size was also evident during late apoptosis in 24–36 h after egg activation. However, the change was not as prominent as that observed in overactivated eggs [8]. It is currently unknown whether the plasma membrane of apoptotic frog eggs sustains critical damage towards the end of apoptosis.

Thus, in a nutshell, irreversible cortical contraction, non-compensated elevation of intracellular calcium, infringement of the plasma membrane, robust depletion of intracellular ATP, inhibition of protein synthesis, and the rapid and dramatic increase in the cell size are the major features that characterize the necrotic process in overactivated frog eggs and distinguish it from other types of cell death.

## 6. Physiological Relevance of Egg Overactivation

Thus, the vast majority of mature unfertilized frog eggs undergo apoptosis, while a small minority of eggs die through necrosis (Figure 3). Apparently, the pattern of cell death, either apoptotic or necrotic, is of little significance in the case of eggs deposited outside the frog’s body. However, it was found that quite a few eggs are retained in the genital tract of *Xenopus* frogs for several days following hormone-induced ovulation. Although the majority of *Xenopus* eggs are normally laid out within 10 to 18 h after hCG injection, up to 5% of eggs still remain in the frog’s body for a much longer time [9]. Some factors, like frog aging, falling temperatures, etc., were reported to cause the retention of mature eggs in the uterus [9,85]. The retained eggs were found to degrade in the genital tract mainly by a caspase-dependent apoptotic process [9]. All of the apoptotic features observed in unfertilized spawned eggs were also observed in the eggs retained in the genital tract, suggesting that the same apoptotic program unfolds in water-deposited and body-retained eggs. Considering that apoptosis evolved as a mechanism to diminish the damaging effects of individual cell death on the whole organism, this process may be very important for the elimination of the mature, overripe eggs retained in the frog body after ovulation. It was hypothesized that egg apoptosis accompanies ovulation in species with external fertilization as a normal process to eliminate unfertilized eggs retained in the genital tract after ovulation [3].

Then, what is the physiological relevance of egg overactivation followed by necrotic cell death? Necrosis is recognized as a pathological and uncontrolled process that occurs due to a catastrophic injury. It is almost always associated with highly damaging pathological conditions if it occurs in the body tissues. In our studies, it was found that a very small proportion of eggs retained in the genital tract after hCG injection bear the distinctive morphological futures of overactivated eggs, such as the white coloring and increased cell diameter. These eggs can easily be distinguished by their unique phenotype. In addition, ATP depletion, which represents a hallmark of egg overactivation, was also observed in the retained overactivated eggs [11]. Thus, like spawned frog eggs, the eggs retained in the frog’s genital tract may occasionally undergo spontaneous overactivation and necrosis. Physiological inducers of spontaneous egg activation are currently mostly unknown. However, it was found that mechanical or oxidative stress can greatly increase the frequency of overactivation. Since physical constriction is known to accompany oviposition in different species, it has been suggested that mechanical stress could be a key factor promoting egg overactivation during oviposition in frogs [11].

Therefore, in general, egg overactivation and necrosis in the genital tract should be regarded as undesirable, pathological, harmful, and uncontrollable outcomes. However, how much damage can necrotic eggs cause in the frog’s genital tract? Markedly, the breach of the plasma membrane in overactivated eggs does not lead to the immediate and massive release of intracellular content, and the eggs maintain their shape and size for some time. This can be explained by the presence of the jelly layer acquired in the genital tract and the hardening of the plasma membrane during oocyte maturation. Furthermore, overactivation can take place only in meiotically-arrested eggs that mature several hours after hormonal stimulation. By that time, the eggs leave the ovaries and advance to a lower part of the reproductive system. In the lower genital tract, the ovisac or uterus stores the eggs temporarily before their deposition outside the frog’s body. The ovisac opens into the cloaca and forms the oviductal sinus [86], providing a common pathway for excretion and reproduction. It seems that egg overactivation in the lower genital tract—i.e., in the ovisac and cloaca—cannot wreak much damage to the animal because its contents are expelled as excrement without an inflammatory reaction from the body. Therefore, it can be concluded that while necrotic cell death is generally a very undesirable scenario in a multicellular organism, egg overactivation and necrosis in the frog’s genital tract should inflict only little or no harm to the animal. It would be important to investigate whether overactivation can occur in the upper parts, the pars recta and pars convoluta, of the oviduct.

Then, is overactivation completely uncontrollable? The findings that mechanical or oxidative stress can greatly increase the frequency of overactivation, especially in aging eggs, indicate that stress intensity and egg condition may significantly affect the proportion of overactivated eggs in natural egg populations. Therefore, minimizing different stresses and egg conditioning could be the strategies to reduce overactivation. Furthermore, it seems that overactivation-triggered cell death unfolds as a sequential and ordered process, and at least the initial step of the overactivation-triggered cell death, such as the elevation of intracellular calcium, can be controlled. Indeed, egg overactivation was shown to be, in part, a calcium-dependent process, which can be attenuated in the presence of calcium chelators [72]. Evidently, some calcium-independent mechanisms also contribute to this process because the inhibition of egg overactivation by both cell-permeable (BAPTA-AM) and impermeable (BAPTA) selective calcium chelators, as well as by their combination, is only partial [72]. Based on the above findings, the approaches that can prevent or attenuate overactivation should be pursued with the aim of increasing egg quality and stability.

Finally, it is currently unknown if mammalian eggs can undergo overactivation. However, if so, the findings in frogs can possibly be applied to mammalian eggs for use in assisted reproduction. Previous studies have not distinguished between activation and overactivation, and morphological markers of overactivation have not been identified in mammalian eggs. The major morphological and cytological features of mammalian egg activation, such as cortical granule exocytosis, zona pellucida hardening, pronucleus formation, and the extrusion of the second polar body [2] should be re-examined in these cells to discriminate between egg activation and overactivation. Also, the intracellular molecular events of egg activation, such as elevation of intracellular calcium, activation of CaMKII, cyclin degradation, ATP reduction, etc., should be confirmed in overactivated mammalian eggs. Distinguishing egg activation and overactivation is very important because the two processes lead to completely distinct, apoptotic or necrotic cell death scenarios that can differently affect body homeostasis.

## Figures and Tables

**Table 1:** Main features of egg overactivation.

Stimulus		Oxidative Stress	Mechanical Stress	Spontaneous Overactivation
Features				
Irreversible cortical contraction		+	+	+
Non-compensted rise of intracellular calcium		+	?	?
Cyclin degradation		+	+	+
MAPK dephosphorylation		?	−	+
ATP depletion		+	+	+
Increase in ADP/ATP ratio		?	+	+
Leakage of ATP and ADP		?	+	+
Termination of protein synthesis		+	?	?
Lipofuscin accumulation		+	?	?
Decline of MMP		+	?	?
Endosomal acidification		+	?	?
Decrease in soluble protein content		+	?	?
Increase in egg size		+	+	+
Caspase activation		−	−	−

The symbols (+) and (−) indicate detected and undetected features of egg overactivation, respectively, and (?) refers to unexamined traits.

## References

[1] Miao Y.-L., Kikuchi K., Sun Q.-Y., Schatten H. (2009). Oocyte aging: Cellular and molecular changes, developmental potential and reversal possibility. Hum. Reprod. Update.

[2] Prasad S., Tiwari M., Koch B., Chaube S.K. (2015). Morphological, cellular and molecular changes during postovulatory egg aging in mammals. J. Biomed. Sci..

[3] Tokmakov A.A., Sato K.I., Stefanov V.E. (2018). Postovulatory cell death: Why eggs die via apoptosis in biological species with external fertilization. J. Reprod. Dev..

[4] Perez G.I., Tao X.J., Tilly J.L. (1999). Fragmentation and death (a.k.a. apoptosis) of ovulated oocytes. Mol. Hum. Reprod..

[5] Tripathi A., Chaube S.K. (2012). High cytosolic free calcium level signals apoptosis through mitochondria-caspase mediated pathway in rat eggs cultured in vitro. Apoptosis.

[6] Houel-Renault L., Philippe L., Piquemal M., Ciapa B. (2013). Autophagy is used as a survival program in unfertilized sea urchin eggs that are destined to die by apoptosis after inactivation of MAPK1/3 (ERK2/1). Autophagy.

[7] Philippe L., Tosca L., Zhang W.L., Piquemal M., Ciapa B. (2014). Different routes lead to apoptosis in unfertilized sea urchin eggs. Apoptosis.

[8] Tokmakov A.A., Iguchi S., Iwasaki T., Fukami Y. (2011). Unfertilized frog eggs die by apoptosis following meiotic exit. BMC Cell Biol..

[9] Iguchi S., Iwasaki T., Fukami Y., Tokmakov A.A. (2013). Unlaid Xenopus eggs degrade by apoptosis in the genital tract. BMC Cell Biol..

[10] Du Pasquier D., Dupré A., Jessus C. (2011). Unfertilized Xenopus eggs die by Bad-dependent apoptosis under the control of Cdk1 and JNK. PLoS ONE.

[11] Tokmakov A.A., Teranishi R., Sato K.I. (2024). Spontaneous Overactivation of Xenopus Frog Eggs Triggers Necrotic Cell Death. Int. J. Mol. Sci..

[12] Masui Y., Markert C.L. (1971). Cytoplasmic control of nuclear behavior during meiotic maturation of frog oocytes. J. Exp. Zool..

[13] Masui Y. (1974). A cytostatic factor in amphibian oocytes: Its extraction and partial characterization. J. Exp. Zool..

[14] Sagata N., Watanabe N., vande Woude G.F., Ikawa Y. (1989). The c-mos proto-oncogene product is a cytostatic factor responsible for meiotic arrest in vertebrate eggs. Nature.

[15] Mueller P.R., Coleman T.R., Kumagai A., Dunphy W.G. (1995). Myt1: A membrane-associated inhibitory kinase that phosphorylates Cdc2 on both threonine-14 and tyrosine-15. Science.

[16] Murakami M.S., vande Woude G.F. (1998). Analysis of the early embryonic cell cycles of Xenopus; regulation of cell cycle length by Xe-wee1 and Mos. Development.

[17] Nakajo N., Yoshitome S., Iwashita J., Iida M., Uto K., Ueno S., Okamoto K., Sagata N. (2000). Absence of Wee1 ensures the meiotic cell cycle in Xenopus oocytes. Genes Dev..

[18] Duckworth B.C., Weaver J.S., Ruderman J.V. (2002). G2 arrest in Xenopus oocytes depends on phosphorylation of cdc25 by protein kinase A. Proc. Natl. Acad. Sci. USA.

[19] Castro A., Peter M., Magnaghi-Jaulin L., Vigneron S., Galas S., Lorca T., Labbé J.C. (2001). Cyclin B/cdc2 induces c-Mos stability by direct phosphorylation in Xenopus oocytes. Mol. Biol. Cell..

[20] Howard E.L., Charlesworth A., Welk J., MacNicol A.M. (1999). The mitogen-activated protein kinase signaling pathway stimulates mos mRNA cytoplasmic polyadenylation during Xenopus oocyte maturation. Mol. Cell. Biol..

[21] Palmer A., Gavin A.C., Nebreda A.R. (1998). A link between MAP kinase and p34(cdc2)/cyclin B during oocyte maturation: p90(rsk) phosphorylates and inactivates the p34(cdc2) inhibitory kinase Myt1. EMBO J..

[22] Mueller P.R., Coleman T.R., Dunphy W.G. (1995). Cell cycle regulation of a Xenopus wee1-like kinase. Mol. Biol. Cell..

[23] Schwab M.S., Roberts B.T., Gross S.D., Tunquist B.J., Taieb F.E., Lewellyn A.L., Maller J.L. (2001). Bub1 is activated by the protein kinase p90 (Rsk) during Xenopus oocyte maturation. Curr. Biol..

[24] Nishiyama T., Ohsumi K., Kishimoto T. (2007). Phosphorylation of Erp1 by p90rsk is required forcytostatic factor arrest in Xenopus laevis eggs. Nature.

[25] Inoue D., Ohe M., Kanemori Y., Nobui T., Sagata N. (2007). A direct link of the Mos–MAPK pathway to Erp1/Emi2 in meiotic arrest of Xenopus laevis eggs. Nature.

[26] Tung J.J., Padmanabhan K., Hansen D.V., Richter J.D., Jackson P.K. (2007). Translational unmasking of Emi2 directs cytostatic factor arrest in meiosis II. Cell Cycle.

[27] Schmidt A., Duncan P.I., Rauh N.R., Sauer G., Fry A.M., Nigg E.A., Mayer T.U. (2005). Xenopus polo-like kinase Plx1 regulates XErp1, a novel inhibitor of APC/C activity. Genes Dev..

[28] Tung J.J., Hansen D.V., Ban K.H., Loktev A.V., Summers M.K., Adler J.R., Jackson P.K. (2005). A role for the anaphase-promoting complex inhibitor Emi2/XErp1, a homolog of early mitotic inhibitor 1, in cytostatic factor arrest of Xenopus eggs. Proc. Natl. Acad. Sci. USA.

[29] Shoji S., Yoshida N., Amanai M., Ohgishi M., Fukui T., Fujimoto S., Nakano Y., Kajikawa E., Perry A.C. (2006). Mammalian Emi2 mediates cytostatic arrest and transduces the signal for meiotic exit via Cdc20. EMBO J..

[30] Musacchio A. (2015). The molecular biology of spindle assembly checkpoint signaling dynamics. Curr. Biol..

[31] Santaguida S., Vernieri C., Villa F., Ciliberto A., Musacchio A. (2011). Evidence that Aurora B is implicated in spindle checkpoint signalling independently of error correction. EMBO J..

[32] Blengini C.S., Nguyen A.L., Aboelenain M., Schindler K. (2021). Age-dependent integrity of the meiotic spindle assembly checkpoint in females requires Aurora kinase B. Aging Cell.

[33] Abrieu A., Brassac T., Galas S., Fisher D., Labbé J.C., Dorée M. (1998). The Polo-like kinase Plx1 is a component of the MPF amplification loop at the G2/M-phase transition of the cell cycle in Xenopus eggs. J. Cell Sci..

[34] Gavin A.C., Ni Ainle A., Chierici E., Jones M., Nebreda A.R. (1999). A p90(rsk) mutant constitutively interacting with MAP kinase uncouples MAP kinase from p34(cdc2)/cyclin B activation in Xenopus oocytes. Mol. Biol. Cell.

[35] Kumagai A., Dunphy W.G. (1996). Purification and molecular cloning of Plx1, a Cdc25-regulatory kinase from Xenopus egg extracts. Science.

[36] Qian Y.W., Erikson E., Maller J.L. (1998). Purification and cloning of a protein kinase that phosphorylates and activates the polo-like kinase Plx1. Science.

[37] Mochida S., Ikeo S., Gannon J., Hunt T. (2009). Regulated activity of PP2A-B55 delta is crucial for controlling entry into and exit from mitosis in Xenopus egg extracts. EMBO J..

[38] Yu J., Zhao Y., Li Z., Galas S., Goldberg M.L. (2006). Greatwall kinase participates in the Cdc2 autoregulatory loop in Xenopus egg extracts. Mol. Cell..

[39] Castilho P.V., Williams B.C., Mochida S., Zhao Y., Goldberg M.L. (2009). The M phase kinase Greatwall (Gwl) promotes inactivation of PP2A/B55delta, a phosphatase directed against CDK phosphosites. Mol. Biol. Cell..

[40] Vigneron S., Brioudes E., Burgess A., Labbé J.C., Lorca T., Castro A. (2009). Greatwall maintains mitosis through regulation of PP2A. EMBO J..

[41] Mochida S., Maslen S.L., Skehel M., Hunt T. (2010). Greatwall phosphorylates an inhibitor of protein phosphatase 2A that is essential for mitosis. Science.

[42] Gharbi-Ayachi A., Labbé J.C., Burgess A., Vigneron S., Strub J.M., Brioudes E., Van-Dorsselaer A., Castro A., Lorca T. (2010). The substrate of Greatwall kinase, Arpp19, controls mitosis by inhibiting protein phosphatase 2A. Science.

[43] Hara M., Abe Y., Tanaka T., Yamamoto T., Okumura E., Kishimoto T. (2012). Greatwall kinase and cyclin B-Cdk1 are both critical constituents of M-phase-promoting factor. Nat. Commun..

[44] Dupré A., Buffin E., Roustan C., Nairn A.C., Jessus C., Haccard O. (2013). The phosphorylation ofARPP19 by Greatwall renders the auto-amplification of MPF independently of PKA in Xenopus oocytes. J. Cell Sci..

[45] De Moor C.H., Richter J.D. (1997). The Mos pathway regulates cytoplasmic polyadenylation in Xenopus oocytes. Mol. Cell. Biol..

[46] Whitaker M. (1996). Control of meiotic arrest. Rev. Reprod..

[47] Stricker S.A. (1999). Comparative biology of calcium signaling during fertilization and egg activation in animals. Dev. Biol..

[48] Ramos I., Wessel G.M. (2013). Calcium pathway machinery at fertilization in echinoderms. Cell Calcium.

[49] Stein P., Savy V., Williams A.M., Williams C.J. (2020). Modulators of calcium signalling at fertilization. Open Biol..

[50] Miao Y.L., Stein P., Jefferson W.N., Padilla-Banks E., Williams C.J. (2012). Calcium influx-mediated signaling is required for complete mouse egg activation. Proc. Natl. Acad. Sci. USA.

[51] Xu Y.R., Yang W.X. (2017). Calcium influx and sperm-evoked calcium responses during oocyte maturation and egg activation. Oncotarget.

[52] Runft L.L., Watras J., Jaffe L.A. (1999). Calcium release at fertilization of Xenopus eggs requires type I IP(3) receptors, but not SH2 domain-mediated activation of PLCgamma or G(q)-mediated activation of PLCβ. Dev. Biol..

[53] Dupont G., Goldbeter A. (1994). Properties of intracellular Ca^2+^ waves generated by a model based on Ca^2+^-induced Ca^2+^ release. Biophys. J..

[54] Wagner J., Li Y.X., Pearson J., Keizer J. (1998). Simulation of the fertilization Ca^2+^ wave in Xenopus laevis eggs. Biophys. J..

[55] Chebotareva T., Taylor J., Mullins J.J., Wilmut I. (2011). Rat eggs cannot wait: Spontaneous exit from meiotic metaphase-II arrest. Mol. Reprod. Dev..

[56] Premkumar K.V., Chaube S.K. (2014). RyR channel-mediated increase of cytosolic free calcium level signals cyclin B1 degradation during abortive spontaneous egg activation in rat. Vitr. Cell. Dev. Biol. Anim..

[57] Xu Z., Abbott A., Kopf G.S., Schultz R.M., Ducibella T. (1997). Spontaneous activation of ovulated mouse eggs: Time-dependent effects on M-phase exit, cortical granule exocytosis, maternal messenger ribonucleic acid recruitment, and inositol 1,4,5-trisphosphate sensitivity. Biol. Reprod..

[58] Ma W., Zhang D., Hou Y., Li Y.H., Sun Q.Y., Sun X.F., Wang W.H. (2005). Reduced expression of MAD2, BCL2, and MAP kinase activity in pig oocytes after in vitro aging are associated with defects in sister chromatid segregation during meiosis II and embryo fragmentation after activation. Biol. Reprod..

[59] Santos H.B., Sato Y., Moro L., Bazzoli N., Rizzo E. (2008). Relationship among follicular apoptosis, integrin beta1 and collagen type IV during early ovarian regression in the teleost Prochilodus argenteus after induced spawning. Cell Tissue Res..

[60] Rauh N.R., Schmidt A., Bormann J., Nigg E.A., Mayer T.U. (2005). Calcium triggers exit from meiosis II by targeting the APC/C inhibitor XErp1 for degradation. Nature.

[61] Hansen D.V., Tung J.J., Jackson P.K. (2006). CaMKII and polo-like kinase 1 sequentially phosphorylate the cytostatic factor Emi2/XErp1 to trigger its destruction and meiotic exit. Proc. Natl. Acad. Sci. USA.

[62] Liu J., Maller J.L. (2005). Calcium elevation at fertilization coordinates phosphorylation of XErp1/Emi2 by Plx1 and CaMK II to release metaphase arrest by cytostatic factor. Curr. Biol..

[63] Wu J.Q., Kornbluth S. (2008). Across the meiotic divide—CSF activity in the post-Emi2/XErp1 era. J. Cell Sci..

[64] Chung E., Chen R.H. (2003). Phosphorylation of Cdc20 is required for its inhibition by the spindle checkpoint. Nat. Cell Biol..

[65] Ferrell J.E. (2002). Self-perpetuating states in signal transduction: Positive feedback, double-negative feedback and bistability. Curr. Opin. Cell Biol..

[66] Nishizawa M., Furuno N., Okazaki K., Tanaka H., Ogawa Y., Sagata N. (1993). Degradation of Mos by the N-terminal proline (Pro2)-dependent ubiquitin pathway on fertilization of Xenopus eggs: Possible significance of natural selection for Pro2 in Mos. EMBO J..

[67] Tokmakov A.A., Stefanov V.E., Iwasaki T., Sato K., Fukami Y. (2014). Calcium signaling and meiotic exit at fertilization in Xenopus egg. Int. J. Mol. Sci..

[68] Levasseur M., Dumollard R., Chambon J.P., Hebras C., Sinclair M., Whitaker M., McDougall A. (2013). Release from meiotic arrest in ascidian eggs requires the activity of two phosphatases but not CaMKII. Development.

[69] Madgwick S., Levasseur M., Jones K.T. (2005). Calmodulin-dependent protein kinase II, and not protein kinase C, is sufficient for triggering cell-cycle resumption in mammalian eggs. J. Cell Sci..

[70] Suzuki T., Suzuki E., Yoshida N., Kubo A., Li H., Okuda E., Amanai M., Perry A.C. (2010). Mouse Emi2 as a distinctive regulatory hub in second meiotic metaphase. Development.

[71] Tokmakov A.A., Awamura M., Sato K.I. (2019). Biochemical Hallmarks of Oxidative Stress-Induced Overactivation of Xenopus Eggs. Biomed. Res. Int..

[72] Tokmakov A.A., Morichika Y., Teranishi R., Sato K.I. (2022). Oxidative Stress-Induced Overactivation of Frog Eggs Triggers Calcium-Dependent Non-Apoptotic Cell Death. Antioxidants.

[73] Sato K., Ogawa K., Tokmakov A.A., Iwasaki T., Fukami Y. (2001). Hydrogen peroxide induces Src family tyrosine kinase-dependent activation of Xenopus eggs. Dev. Growth Differ..

[74] Sato K., Tokmakov A.A., Iwasaki T., Fukami Y. (2000). Tyrosine kinase-dependent activation of phospholipase Cgamma is required for calcium transient in Xenopus egg fertilization. Dev. Biol..

[75] Nikoletopoulou V., Markaki M., Palikaras K., Tavernarakis N. (2013). Crosstalk between apoptosis, necrosis and autophagy. Biochim. Biophys. Acta.

[76] Kung G., Konstantinidis K., Kitsis R.N. (2011). Programmed necrosis, not apoptosis, in the heart. Circ. Res..

[77] Galluzzi L., Vitale I., Aaronson S.A., Abrams J.M., Adam D., Agostinis P., Alnemri E.S., Altucci L., Amelio I., Andrews D.W. (2018). Molecular mechanisms of cell death: Recommendations of the Nomenclature Committee on Cell Death 2018. Cell Death Differ..

[78] Xiong W., Ferrell J.E. (2003). A positive-feedback-based bistable memory module’ that governs a cell fate decision. Nature.

[79] Watanabe N., Hunt T., Ikawa Y., Sagata N. (1991). Independent inactivation of MPF and cytostatic factor (Mos) upon fertilization of Xenopus eggs. Nature.

[80] Eguchi Y., Shimizu S., Tsujimoto Y. (1997). Intracellular ATP levels determine cell death fate by apoptosis or necrosis. Cancer Res..

[81] Huang F., Vemuri M.C., Schneider J.S. (2004). Modulation of ATP levels alters the mode of hydrogen peroxide-induced cell death in primary cortical cultures: Effects of putative neuroprotective agents. Brain Res..

[82] Miyoshi N., Watanabe E., Osawa T., Okuhira M., Murata Y., Ohshima H., Nakamura Y. (2008). ATP depletion alters the mode of cell death induced by benzyl isothiocyanate. Biochim. Biophys. Acta.

[83] Barros L.F., Hermosilla T., Castro J. (2001). Necrotic volume increase and the early physiology of necrosis. Comp. Biochem. Physiol. A Mol. Integr. Physiol..

[84] Karch J., Molkentin J.D. (2015). Regulated necrotic cell death: The passive aggressive side of Bax and Bak. Circ. Res..

[85] Witschi E. (1952). Overripeness of the egg as a cause of twinning and teratogenesis: A review. Cancer Res..

[86] Méndez-Tepepa M., Morales-Cruz C., García-Nieto E., Anaya-Hernández A. (2023). A review of the reproductive system in anuran amphibians. Zool. Lett..

